# The longitudinal associations between filial piety belief and mobile phone addiction: from the perspective of self-determination theory

**DOI:** 10.3389/fpsyg.2025.1635463

**Published:** 2025-09-18

**Authors:** Hua Wei, Jiajia Tang, Hemuqing Xu, Juan Shi, Qiufang Wu, Yuancai Song

**Affiliations:** ^1^Department of Psychology, School of Education Science, Qingdao University, Qingdao, China; ^2^School of Education Science, Xinyang Normal University, Xinyang, China

**Keywords:** filial piety beliefs, basic needs satisfaction, ego-depletion, mobile phone addiction, self-determination theory

## Abstract

Based on the dual filial piety model, this study aims to examine the impact of filial piety beliefs on mobile phone addiction. Drawing on self-determination theory, it explores the mediating effect of basic need satisfaction and tests the moderating effect of ego-depletion. Through a 6-month longitudinal study, a total of 1,468 adolescents completed the questionnaires, which included the dual filial piety scale, basic need satisfaction scale, ego-depletion scale, and mobile phone addiction scale. The results showed that reciprocal filial piety belief has a negative impact on mobile phone addiction, while authoritarian filial piety belief has no significant effect on mobile phone addiction. In addition, reciprocal filial piety belief influences mobile phone addiction through the mediating effect of basic needs satisfaction. Reciprocal filial piety belief enhances basic needs satisfaction, which in turn decreases the likelihood of developing mobile phone addiction. Moreover, the effect of basic need satisfaction on mobile phone addiction is moderated by ego-depletion. Specifically, for adolescents with low levels of ego-depletion, basic need satisfaction can reduce mobile phone addiction. In contrast, for adolescents with high levels of ego-depletion, basic need satisfaction has no significant effect on mobile phone addiction.

## 1 Introduction

Mobile phones have become an indispensable part of adolescents' daily life, permeating all aspects of their learning, social interactions, and entertainment. Although mobile phones can help adolescents increase knowledge, expand social interaction, and obtain happiness, they can also easily lead to addiction. Adolescent mobile phone addiction is influenced by individual, family, school, and societal factors (de Freitas et al., 2021; De-Sola Gutiérrez et al., 2016; Sahu et al., 2019). In terms of family factors, parenting styles, parental monitoring, parental support, and parental conflicts can all affect mobile phone addiction (de Freitas et al., 2021; De-Sola Gutiérrez et al., 2016; Sahu et al., 2019). Despite their significant theoretical and practical contributions, these studies have overlooked the role children in family factors on mobile phone addiction.

In traditional Chinese society, children play an active role in the family. The concept of filial piety reflects the normative roles of children within the family. Given that parental roles can impact mobile phone addiction, it is worth investigating whether filial piety beliefs, which reflect children's roles, also influence mobile phone addiction. Previous research has demonstrated that filial piety beliefs are closely linked to adolescents' self-concept, interpersonal skills, and emotional-cognitive characteristics (Bedford and Yeh, 2020), all of which can impact mobile phone addiction (de Freitas et al., 2021; De-Sola Gutiérrez et al., 2016; Sahu et al., 2019). Therefore, we hypothesize that filial piety beliefs may influence mobile phone addiction.

## 2 Hypotheses development

### 2.1 Filial piety and mobile phone addiction

Filial piety is a set of ethical principles that shape the relationship between children and their parents (Bedford and Yeh, 2020). The dual filial piety model categorizes filial piety into two types: authoritarian filial piety (AFP) and reciprocal filial piety (RFP). AFP is based on the Confucian principle of ‘respect the respected', emphasizing the role norms in a hierarchical society and the unconditional obedience of subordinates to superiors. RFP is based on the Confucian principle of “close to the closest,” posits that the closeness of familial bonds shapes the intensity of relationships.

Empirical studies have consistently demonstrated that individuals with stronger beliefs in RFP tend to have greater psychological resources and demonstrate better adaptive capabilities, while AFP tends to have the opposite effect (Bedford and Yeh, 2019; Han and Cheung, 2024; Wei et al., 2022; Wei and Liu, 2022). Based on resource conservation theory and related studies, the more resources individuals have, the better their adaptation will be. When resources are insufficient, they seek compensation, which may lead to adaptation problems (Hobfoll, 1989). RFP belief is positively correlated with higher self-esteem, enhanced interpersonal competencies, and reduced cognitive distortions and negative emotional experiences, while AFP generally produces the opposite outcomes (Bedford and Yeh, 2020). Given that mobile phones are convenient and multifunctional, adolescents with insufficient psychological resources may use them as a form of compensation. Excessive reliance on this compensation can result in mobile phone addiction.

Therefore, we propose Hypothesis 1 (H1): Reciprocal filial piety negatively affects mobile phone addiction, while authoritarian filial piety positively affects mobile phone addiction.

### 2.2 The mediating role of basic psychological needs satisfaction

According to self-determination theory, individual characteristics can influence basic psychological needs, which in turn affect an individual's adaptability (Ryan and Deci, 2017). Therefore, we suggest that filial piety belief affects the satisfaction of fundamental psychological needs, which subsequently impacts mobile phone addiction.

First, filial piety belief may affect the fulfillment of basic psychological needs. Self-determination theory asserts that the social environment is a primary factor influencing the satisfaction of individual needs, with the interpersonal environment being particularly crucial (Ryan and Deci, 2017). A supportive and harmonious interpersonal environment promotes the fulfillment of basic psychological needs. RFP, which emphasizes close interaction with parents, can develop a stronger ability for perspective-taking and reduce hostile attribution (Wei and Liu, 2022). Consequently, it helps children gain more social support and fulfill their basic psychological needs. In contrast, AFP demands that children show absolute obedience to their parents, which reduces perspective-taking, thereby diminishing their ability to understand others' intentions. The difficulty in understanding underlying connotations leads to negative assumptions about others' social behavior, fueling conflicts and hindering the fulfillment of psychological needs.

Secondly, fulfilling basic psychological needs can reduce smartphone addiction. When these needs (autonomy, relatedness, competence) remain unmet, adolescents often turn to smartphones for compensation (Ryan and Deci, 2017). Given that smartphones can provide autonomy (through choice of content), relatedness (via social apps), and competence (through gaming achievements), they create a reliance that may lead to addiction through negative reinforcement.

Therefore, we propose Hypothesis H2: Filial piety beliefs influence smartphone addiction via psychological needs satisfaction. Reciprocal filial piety enhances psychological needs satisfaction, reducing the risk of addiction, whereas authoritarian filial piety undermines psychological needs satisfaction, increasing the risk of addiction.

### 2.3 The moderating effect of ego-depletion

While unmet needs may “push” individuals toward compulsive use, compelling external rewards (e.g., social validation, gaming achievements) may “pull” them in even more strongly (Berridge and Robinson, 2016). When these external incentives dominate, the protective effect of need satisfaction may weaken.

Smartphones provide adolescents with diverse activities and rich content, which can easily induce a state of flow and craving during use (Khang et al., 2013). In other words, the temptation of mobile phones as an external factor is common among adolescents. When confronted with this temptation, insufficient self-control resources (ego depletion) increase the likelihood of addiction (Baumeister and Vonasch, 2015). When adolescents experience a high degree of ego-depletion, they lack sufficient willpower to resist the temptation of mobile phones. Under such circumstances, external temptation may become a dominant factor contributing to mobile phone addiction, while the influence of internal psychological needs satisfaction on addiction may be diminished. In essence, even though adolescents' fundamental psychological needs are satisfied, insufficient self-control resulting from a state of ego-depletion, makes them more susceptible to the allure of mobile phones, thereby increasing their risk of addiction.

Therefore, this study proposed Hypothesis H3: The effect of basic psychological needs satisfaction on mobile phone addiction is moderated by ego-depletion. Specifically, Adolescents with high levels of ego depletion experience a weaker effect of basic need satisfaction on mobile phone addiction compared to those with low levels of ego depletion.

## 3 Methods

### 3.1 Participants

Participants were selected from two middle schools in Henan Province, and the questionnaires were administered during class sessions. Informed consent was obtained from all participants and their legal guardians prior to the surveys. At the first wave, measures of filial piety belief, basic psychological needs satisfaction, and mobile phone addiction were collected from 1,727 participants. At the second wave, measures of basic psychological needs satisfaction, ego-depletion, and mobile phone addiction were collected from 1,681 participants. The interval between the two waves was 6 months. After excluding missing and patterned responses, a total of 1,468 valid questionnaires were retained, resulting in a valid response rate of 90.23%. Among the participants, 717 were boys and 751 were girls; 403 were in the first year, 763 in the second year, and 302 in the third year of middle school.

### 3.2 Measures

#### 3.2.1 Filial piety belief

The filial piety belief scale was developed from the original Filial Piety Scale (Wei et al., 2022). This scale have been previously validated on a Chinese adolescent sample (Jin et al., 2019). This scale encompasses two dimensions: reciprocal filial piety and authoritarian filial piety, totaling 10 items. Participants responded on a 5-point Likert scale, where higher scores denote stronger beliefs in filial piety. In this study, the Cronbach alpha coefficients were 0.82 for RFP and 0.73 for AFP at T1.

#### 3.2.2 Basic psychological needs satisfaction

In this study, we employed the Basic Psychological Needs Satisfaction Scale to assess participants' satisfaction of basic needs (Wei et al., 2022). This scale has been previously validated in a Chinese adolescent population (Ti et al., 2025). The scale comprises 9 items, with responses given on a 7-point Likert scale, where higher scores indicate greater satisfaction of these fundamental needs. The Cronbach's alpha coefficient for basic need satisfaction was 0.90 at Time 1 (T1) and 0.92 at Time 2 (T2).

#### 3.2.3 Ego-depletion

The ego-depletion scale evaluates the extent to which individuals experience a reduction in their willpower after engaging in tasks that require self-control (Lanaj et al., 2014). This scale has been previously validated on a Chinese adolescent sample (He et al., 2022). This scale comprises five items and uses a 7-point Likert scoring system. A higher total score indicates more ego depletion. In this study, the Cronbach's alpha coefficient for ego depletion at T2 was 0.89.

#### 3.2.4 Mobile phone addiction

Mobile phone addiction was measured using the mobile phone addiction scale (Hong et al., 2012). This scale have been previously validated on a Chinese adolescent sample (Xia et al., 2024). This scale comprises 11 items and uses a 6-point Likert-type scoring system. A higher total score indicates a greater degree of mobile phone addiction. In this study, the Cronbach's alpha coefficient for mobile phone addiction was 0.88 at T1 and 0.90 at T2.

## 4 Result

Descriptive statistics were computed using SPSS 21. Mediating and moderating effects were analyzed using the PROCESS 3.0 macro for SPSS.

### 4.1 Correlation coefficients

Correlation analysis was conducted using the mean scores of each variable, with detailed results presented in Table 1.

**Table 1:** The means, standard deviations and correlation coefficients (*n* = 1,468).

**Variables**	***M***	***SD***	**1**	**2**	**3**	**4**	**5**	**6**	**7**
1. Reciprocal filial piety T1	4.71	0.50	—						
2. Authoritarian filial piety T1	2.64	0.86	0.28^**^	—					
3. Basic need satisfactionT1	5.48	1.07	0.35^**^	0.15^**^	—				
4. Basic need satisfactionT2	5.31	1.34	0.23^**^	0.11^**^	0.45^**^	—			
5. Mobile phone addiction T1	3.08	1.18	−0.18^**^	−0.08^**^	−0.19^**^	−0.16^**^	—		
6. Mobile phone addiction T2	3.11	1.18	−0.16^**^	−0.03	−0.13^**^	−0.16^**^	0.53^**^	—	
7. Ego-depletion T2	2.07	0.76	−0.19^**^	−0.11^**^	−0.22^**^	−0.28^**^	0.30^**^	0.40^**^	—

** *p* < 0.01.

### 4.2 Reciprocal filial piety and mobile phone addiction: a moderated mediating model

The moderated mediating model was analyzed in two steps. First, a mediating model was examined. Subsequently, the moderated mediating model was tested using the deviation-corrected non-parametric percentile Bootstrap procedure with 5,000 samples to calculate 95% confidence intervals. This analysis was conducted using Models 4 and 14 of the PROCESS macro. All independent variables, mediating variables, and moderating variables were standardized during the analysis, controlling for gender and age. For simplicity, the regression coefficients were not presented.

The results of mediation analysis (Model 4) are presented in Table 2. RFP at T1 significantly negatively predicted mobile phone addiction at T2 (β = −0.07, *p* < 0.01; Equation 1. Additionally, RFP at T1 positively predicted basic need satisfaction at T2 (β = 0.07, *p* < 0.01; Equation 2. When both RFP at T1 and basic need satisfaction at T2 were included in the model, the effect of RFP at T1 on mobile phone addiction at T2 remained significant (β = −0.06, *p* < 0.05), and basic need satisfaction at T2 negatively predicted mobile phone addiction at T2 (β = −0.07, *p* < 0.01; Equation 3). The indirect effect size (*ab*) was −0.01, with a bias-corrected bootstrap standard error (*SE*) of 0.003. The 95% confidence interval for the indirect effect was (−0.001, −0.013), which does not include zero, indicating a significant mediation effect of basic need satisfaction at T2 between RFP at T1 and mobile phone addiction at T2.

**Table 2:** Regression analysis of the simple mediation model (*n* = 1,468).

**Predictors**	**Equation 1 (criterion: MPA T2)**		**Equation 2 (criterion: BNS T2)**		**Equation 3 (criterion: MPA T2)**	
	***β***	***t***	***β***	***t***	***β***	***t***
Basic need satisfaction T1	−0.01	−0.50	0.43^**^	15.94^**^	−0.02	0.66
Mobile phone addiction T1	0.52	22.83^**^	−0.07	−3.16^**^	0.51	22.57^**^
Reciprocal filial piety T1	−0.07	−2.80^**^	0.07	2.93^**^	−0.06	−2.58^*^
Basic need satisfactionT2					−0.07	−2.92^**^
*R^2^*	0.29		0.47		0.29	
*F*	119.70^**^		82.35^**^		101.68^**^	

* *p* < 0.05,

** *p* < 0.01.

In the second step of the analysis, ego depletion at T2 was entered into the model. As shown in Table 3, the results of the moderated mediating model (model 14) indicated that the interaction term of basic need satisfaction at T2 and ego-depletion at T2 had a significant predictive effect on mobile phone addiction at T2 (β = 0.06, *p* < 0.01). The 95% confidence interval is (0.03, 0.13).

**Table 3:** Regression analysis of moderated mediating model (*n* = 1,468).

**Predictors**	**(Criterion: mobile phone addiction T2)**	
	β	***t***
Basic need satisfaction T1	0.03	1.11
Mobile phone addiction T1	0.45	19.90^**^
Reciprocal filial piety T1	−0.04	−1.76
Basic need satisfaction T2	−0.03	−1.20
Ego-depletion T2	0.26	11.08^**^
Basic need satisfaction T2 × ego-depletion T2	0.06	3.29^**^
*R^2^*	0.35	
*F*	99.73^**^	

** *p* < 0.01.

**Figure 1:**
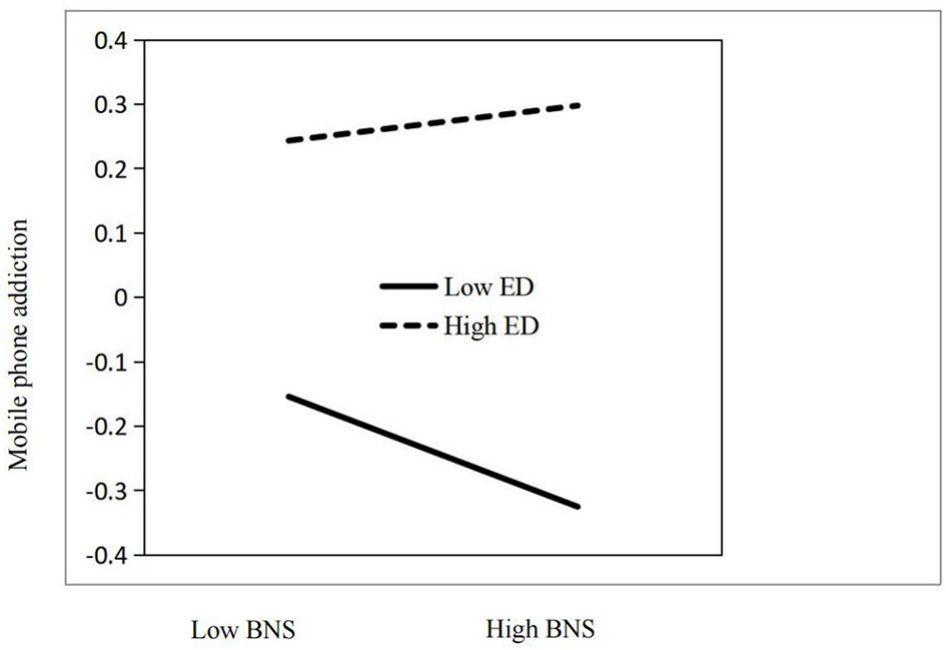
The interaction plot. ED, ego-depletion; BNS, basic needs satisfaction.

To reveal how ego-depletion T2 moderates the effects of basic need satisfactionT2 on mobile phone addiction T2, we conducted a simple effect analysis. The results indicated that when ego-depletion T2 is lower (–*1 SD*), basic need satisfactionT2 had a significant impact on mobile phone addiction T2 (β_simple_ = −0.09, *p* < 0.01); when ego-depletion T2 is higher (+ *1 SD*), basic need satisfactionT2 has no significant effect on T2 addiction (β_*simple*_ = 0.03, *p* > 0.01; Figure 1).

**Figure 2:**
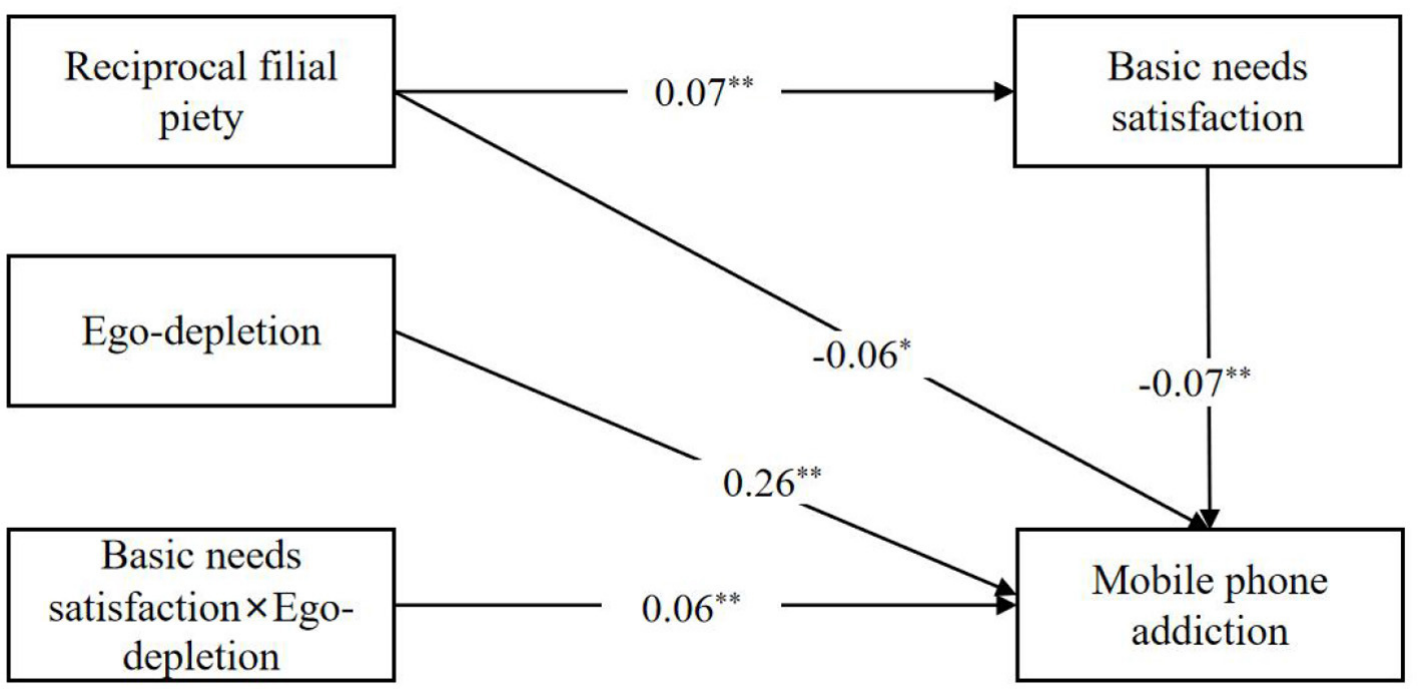
The integrated model. ^*^p < 0.05, **p < 0.01.

According to the results of Model 4 and model 14, we constructed an integrated model (Figure 2).

## 5 Discussion

### 5.1 The impact of filial piety belief on mobile phone addiction in adolescents

This study found that RFP was negatively associated with mobile phone addiction, while AFP was not significantly associated with mobile phone addiction. This is the first study exploring the associations between RFP and mobile phone addiction among adolescents. Specifically, the results indicated that RFP among adolescents is significantly negatively associated with mobile phone addiction, aligning with the research hypothesis and consistent with previous studies in the field of digital addiction (Han and Cheung, 2024; Wan et al., 2024). These results indicate that filial piety, a traditional element of Chinese culture, continues to play a significant role in the digital age.

However, this study did not find a significant association between AFP and mobile phone addiction among adolescents, which differs from previous studies using university students samples. Previous research has shown that AFP can increase the risk of internet addiction, which includes social networking and short video addiction, through the mediating effects of increased maladaptive cognitions and other factors (Fu et al., 2024; Wan et al., 2024; Wei et al., 2019). The age differences among participants in different studies may account for the discrepancies in the findings. For adolescents, AFP may increase the risk of mobile phone addiction through maladaptive cognitions and other pathways. However, it may inhibit addictive behaviors. Therefore, these two opposing mechanisms may lead to an insignificant overall effect. On the one hand, adolescents with high levels of AFP are more likely to adhere to parental guidelines on mobile phone use, thereby regulating their excessive usage. Chinese parents tend to set strict limits on their adolescents' mobile phone use and generally prohibit excessive usage. Compliance with the limits serves as a form of external regulation that helps adolescents resist the temptation of mobile phones. On the other hand, AFP may enhance adolescents' satisfaction of basic psychological needs, which in turn may reduce their craving for mobile phone. With regard to relationship needs, adolescents tend to maintain a relatively close emotional bond with their parents during adolescence, a key period of identity exploration. AFP can effectively meet adolescents' need for relationship by promoting harmonious parent-child interactions and reducing intergenerational conflict. With regard to competence needs, adolescents with higher levels of AFP are more likely to accept their parents' behavioral guidance. Such compliance not only improves the effectiveness of task management but also indirectly enhances needs for competence by fostering a sense of accomplishment through task completion. When adolescents' psychological needs are fully met through real-life family interactions, their motivation to seek compensatory satisfaction through mobile phones can be reduced.

This study examined mobile phone addiction from the perspective of filial piety, offering an alternative to Western theories that focus on the role of parents (e.g., attachment theory) (Bowlby, 1969). While prior research has emphasized parental influences, such as parenting styles, our findings demonstrate that children's filial piety belief also plays a significant role in shaping addiction patterns, highlighting the bidirectional nature of the parent–child dynamic in Chinese culture. This bridges a critical gap in developmental psychology by demonstrating that both parental behaviors and children's perception of their family roles jointly influence adolescent digital behavior, highlighting the need for a balanced examination of both dimensions in future research.

The findings of this study on filial piety and mobile phone addiction have implications beyond China, offering valuable insights for other Confucian-influenced societies—such as Japan, South Korea, and Malaysia—where both phenomena are also prevalent. While filial piety remains a core cultural value across East Asia, its relevance to Western contexts is gaining attention, as reflected in growing scholarly interest in countries, such as Turkey, the United States, and Poland in the areas of intergenerational relationships and social behavior (Lim et al., 2022; Mottram and Horta Su, 2005; Róycka-Tran et al., 2021; Różycka-Tran et al., 2021). Future research should further examine the cross-cultural applicability of filial piety in understanding adolescent digital addiction on a global scale.

### 5.2 The mediating role of basic psychological needs

The present findings contribute to both theoretical development and cultural extension of Self-Determination Theory (SDT; Deci and Ryan, 2000). The original SDT underscores the central role of parents in fulfilling adolescents' basic psychological needs and supporting their adaptation to the social environment. Empirical studies adopting this parent-centered perspective have consistently shown, for example, that parental psychological control predicts maladaptive outcomes —such as smartphone addiction—through its impact on the satisfaction of basic psychological needs (Yao et al., 2022). In contrast, the present study reveals that, within a Confucian cultural framework, adolescents' active internalization of the expectations associated with their family roles —driven by RFP—can also satisfy their basic psychological needs and, in turn, reduce smartphone addiction. This mechanism underscores the adaptive function of cultural value internalization and challenges the prevailing paradigm that views children primarily as passive recipients of parental influence. Consistent with our findings, prior research has demonstrated that RFP predicts lower involvement in cyberbullying through greater need satisfaction (Wei et al., 2022). By delineating the intrinsic connection between internalized cultural values and basic psychological need satisfaction, this study broadens the applicability of SDT beyond Western contexts and offers a culturally grounded framework for understanding adolescent adaptation.

Beyond its theoretical implications, this study also holds practical significance. In contemporary China, the rising popularity of Western parenting philosophies has lead to improvements in parenting styles, which previously lacked support for children's autonomy, while contributing to increased parental anxiety. Excessive parenting anxiety and stress have even contributed to declining marriage and fertility rates among young adults (Höglund and Hildingsson, 2022). Therefore, socialization agents beyond parents, including schools, mass media, and community programs, can play a crucial role in promoting filial piety values. This socially embedded approach not only reduces the exclusive burden on parents but also aligns with the multifaceted pathways—family, educational, and media influences—through which adolescents develop filial piety. The implications of our findings also extend to individualistic Western societies, where children's rights are often emphasized while their familial responsibilities receive comparatively less attention. This imbalance may manifest as diminished respect and care toward parents. A lack of respect for parents may intensify intergenerational conflict and undermine the effectiveness of parental guidance, while insufficient care from children may lead parents to perceive ingratitude, reducing their willingness to offer emotional and material support. Incorporating the principle of RFP into parent–child relationships may help restore this right–responsibility balance, thereby fostering adolescents' need satisfaction and social adaptation. However, this proposition is based on theoretical extrapolation from Chinese samples. Future cross-cultural research is needed to empirically test whether reciprocal filial piety fosters positive developmental outcomes through basic psychological need satisfaction in Western contexts.

### 5.3 The moderating role of ego-depletion

This study found that the impact of basic need satisfaction on mobile phone addiction is moderated by ego-depletion. Specifically, for adolescents with lower levels of ego-depletion, basic need satisfaction can reduce mobile phone addiction. However, for adolescents with high levels of ego-depletion, basic need satisfaction does not impact on mobile phone addiction. Even without an internal need, adolescents with depleted self-control are more vulnerable to external influences, such as stimulating content online, heightening the risk of addiction (Baumeister et al., 2007). These results suggest that research and intervention on adolescent mobile phone addiction should address not only psychological needs but also psychological resources. In practical interventions, preserving and restoring adolescents' resources for self-control is essential for helping them overcome ego-depletion.

## Data Availability

The original contributions presented in the study are included in the article/Supplementary material, further inquiries can be directed to the corresponding author.
