# Supplementary material for: The longitudinal associations between filial piety belief and mobile phone addiction: from the perspective of self-determination theory
Source: Front Psychol. 2025 Sep 18;16:1635463. doi: 10.3389/fpsyg.2025.1635463 (PMC12488399; doi:10.3389/fpsyg.2025.1635463)
Supplement: Supplementary file 1 [file Data_Sheet_1.zip › Date Sheet/Description of Raw Data.docx]

1. **RFP T1** = Reciprocal filial piety Time 1
2. **AFP T1** = Authoritative filial piety Time1
3. **BNS** **T1** = Basic needs satisfaction Time 1
4. **BNS** **T2** = Basic needs satisfaction Time 2
5. **MPA T1** = Mobile phone addiction Time 1
6. **MPA T2** = Mobile phone addiction Time 2
7. **ED T2** = Ego-depletion Time 2

**T1** and **T2** denote different points in time, with **T1** representing the first point in time and **T2** representing the second point in time.
